# The Diagnostic and Prognostic Value of Immunohistochemical Markers in Bladder Cancer: A Real-World Study of 477 Patients

**DOI:** 10.3390/jcm15156124

**Published:** 2026-08-06

**Authors:** Xingxing Tang, Jia Liu, Qiang Zhao, Xiao Yang, Yong Yang, Peng Du

**Affiliations:** Key Laboratory of Carcinogenesis and Translational Research (Ministry of Education), Department of Urology, Peking University Cancer Hospital & Institute, No. 52 Fucheng Road, Haidian District, Beijing 100142, China; xingxing.tang@bjcancer.org (X.T.); liujiauro@bjcancer.org (J.L.); pkuch_zhaoq@bjcancer.org (Q.Z.); x_yang@bjcancer.org (X.Y.); yong.yang@bjcancer.org (Y.Y.)

**Keywords:** bladder cancer, immunohistochemical markers, Uroplakin 3, Ki67, Her2

## Abstract

**Introduction**: To investigate the diagnostic and prognostic value of commonly used immunohistochemical markers regarding high-grade disease, muscle-invasion bladder cancer (MIBC) and survival in bladder cancer. **Methods**: A total of 477 patients diagnosed with bladder cancer at Peking University Cancer Hospital between 2009 and 2024 were included. The immunohistochemical markers included CK7, CK20, GATA3, Uroplakin 3, CK5/6, P40, P63, Syn, CD56, CGA, Vimentin, S100, LCA, Ki67, P53, Her2, EGFR, PD-L1, PD-1 tumor, PD-1 stroma, and Pan-TRK. Logistic analyses were used to determine the diagnostic value of the markers for high-grade disease and MIBC. Cox regression analyses were used to determine the prognostic value of the markers for survival. **Results**: We found Ki67 positivity (*p* < 0.001) and Her2 positivity (*p* = 0.001) might have an association with high-grade disease, while no marker demonstrated diagnostic value for MIBC. Patients with CK7 positivity (*p* = 0.012), Uroplakin 3 positivity (*p* = 0.004), and PD-1 (stroma) positivity (*p* = 0.037) had better overall survival (OS) than those with negative expression, while Cox regression analyses showed only CK7 positivity (*p* = 0.025) and Uroplakin 3 positivity (*p* = 0.008) had prognostic value for better OS. However, considering there were only 2 CK7-negative patients, the sample size might be insufficient to demonstrate its prognostic value. **Conclusions**: These results suggest that Ki67 positivity and Her2 positivity might have association with high-grade disease, and Uroplakin 3 positivity might have prognostic value for better OS. However, these conclusions require further clarification through larger-scale studies in the future.

## 1. Introduction

Bladder urothelial carcinoma (bladder cancer) is one of the most common malignancies [1], with the majority of patients requiring surgical treatment, including transurethral resection of bladder tumor (TURBT) and radical cystectomy (RC) [2]. Postoperative specimens are routinely subjected to immunohistochemical staining, and various markers can indicate tumor aggressiveness and prognosis, as well as guide subsequent therapeutic decision-making, such as Her2 and PD-L1 [3]. Currently, immunohistochemical markers routinely used in clinical practice for bladder cancer encompass multiple categories: epithelial/urothelial markers (CK7, CK20, GATA3, and Uroplakin 3), basal/squamous differentiation markers (CK5/6, P40 and P63), neuroendocrine markers (Syn, CD56 and CGA), mesenchymal/melanocytic markers (Vimentin and S100), lymphohematopoietic markers (LCA), proliferation/prognostic markers (Ki67, P53), therapeutic target markers (Her2 and EGFR), immune checkpoint markers (PD-L1, PD-1 tumor and PD-1 stroma), and Pan-TRK. Most of these clinically utilized markers have been extensively investigated, and many have proven valuable in determining tumor origin and differentiation status [4,5]. Nevertheless, no single study has comprehensively evaluated the diagnostic value of these markers for pathological characteristics and their prognostic value for survival within the same cohort. Therefore, we conducted this study to investigate the diagnostic and prognostic value of the immunohistochemical markers for bladder cancer regarding pathological characteristics and survival, with the aim of providing preliminary insights for future identification of molecular subtypes and survival-predictive markers in bladder cancer.

## 2. Materials and Methods

### 2.1. Study Population

This single-center retrospective study included patients diagnosed with bladder cancer who underwent surgical treatment and obtained pathological results at Peking University Cancer Hospital between 2009 and 2024. Surgical procedures included TURBT and RC. The inclusion criteria were: (1) male or female patients aged over18 years; (2) diagnosis of bladder urothelial carcinoma and underwent surgical treatment, including TURBT and RC; (3) postoperative pathological diagnosis of bladder urothelial cancer. Exclusion criteria included: (1) concomitant other malignancies; (2) urothelial carcinoma mixed with squamous cell carcinoma, adenocarcinoma, neuroendocrine carcinoma, or other components; (3) other conditions deemed inappropriate for inclusion by the investigators.

### 2.2. Study Methods

This was a retrospective study in which we analyzed immunohistochemistry results obtained from the formal pathology reports, and we did not collect additional paraffin-embedded tissue samples for further immunohistochemical staining analysis. Patient demographic information (hospital number, age, sex, etc.) and pathological data (grade, TNM stage, immunohistochemical marker expression, etc.) were collected through the medical record system of Peking University Cancer Hospital. Pathological staging was based on the 8th edition of the American Joint Committee on Cancer (AJCC) TNM staging system [6], and pathological grading was based on the 2022 World Health Organization (WHO) classification [7]. All pathological specimens were interpreted by pathologists from the Department of Pathology at Peking University Cancer Hospital. Following markers were included for analysis: epithelial/urothelial markers (CK7, CK20, GATA3 and Uroplakin 3), basal/squamous differentiation markers (CK5/6, P40 and P63), neuroendocrine markers (Syn, CD56 and CGA), mesenchymal/melanocytic markers (Vimentin and S100), lymphohematopoietic markers (LCA), proliferation/prognostic markers (Ki67 and P53), therapeutic target markers (Her2 and EGFR), immune checkpoint markers (PD-L1, PD-1 tumor and PD-1 stroma), and Pan-TRK.

### 2.3. Immunohistochemical Staining

The immunohistochemical staining in this study followed standard procedures. Paraffin sections were deparaffinized by immersion in Xylene solution and rehydrated with gradient concentration Ethanol. After antigen retrieval of all sections with Citrate buffer pH 6.0 or Tris/EDTA buffer pH 9.0 at 98 °C for one hour, endogenous peroxidase activity was blocked with 3% hydrogen peroxide for 30 min. Then all the sections were blocked with goat serum for 30 min and later incubated overnight with the primary antibodies at 4 °C. The next day, after being treated with the secondary antibody for 1 h, DAB Substrate Kit (Cat# 34065, Thermo Fisher Scientific, Waltham, MA, USA) was used for color development, followed by counterstain with hematoxylin for 1 min, soaking in hydrochloric acid alcohol for 1 s, and turning blue in water for 1 h. After hydration with gradient-concentration ethanol and xylene, neutral resin was used for sealing. After all the sections were completely dried, they were scanned with a digital slide scanner, and SlideViewer v2.6 (3DHISTECH, Budapest, Hungary) was used for image output.

Her2 expression of 2+ and 3+ was defined as Her2 positivity [8], Ki67 expression >20% was defined as high expression (positivity) [9], EGFR expression of 2+ and 3+ was defined as EGFR positivity [10], and CPS > 10 was defined as PD-L1 positivity [11].

### 2.4. Statistical Analysis

Descriptive statistics were used to summarize patient characteristics. Categorical variables were expressed as numbers and percentages, rounded to two decimal places; continuous variables were expressed as mean ± standard deviation (SD), rounded to three decimal places. The Shapiro–Wilk test was used to assess whether data followed a normal distribution. The *t*-test was used for intergroup comparisons of continuous variables with normal distribution, and the Mann–Whitney U test was used for intergroup comparisons of continuous variables with non-normal distribution. Chi-square test was used for intergroup comparison of categorical variables, while Fisher’s exact test was used when expected cell counts were below five. Univariate and multivariate logistic regression analyses were used to evaluate the diagnostic value. The Kaplan–Meier method and log-rank test were used to compare differences in OS and CSS among groups. The Cox proportional hazards regression model was used to analyze whether a marker was risk factor for OS or CSS. Graphs were generated using GraphPad Prism v9.3.1 (GraphPad Software, La Jolla, CA, USA). All statistical analyses were performed using Stata (Version 19.0; StataCorp LLC, College Station, TX, USA). A *p*-value < 0.05 was considered statistically significant.

## 3. Results

### 3.1. Patients’ Characteristics

A total of 477 bladder cancer patients were enrolled in this study. The mean age was 67.405 years, and the mean follow-up time was 43.649 months. 378 patients (79.25%) were male, 371 patients (77.78%) underwent TURBT, and the remaining 106 patients (22.22%) received RC. There were 103 patients (21.59%) with low-grade disease and 374 patients (78.41%) with high-grade disease. Most patients were diagnosed with stage Ta (44.03%) and T1 (32.49%) disease, while 106 patients (22.22%) had muscle-invasive bladder cancer (MIBC). Most patients (80.41%) had no lymph node metastasis. Survival events occurred in 73 patients (15.30%), with a mean survival time of 27.658 months, while the remaining 404 patients (84.70%) did not experience survival events. The characteristics of the patients are presented in Table 1.

### 3.2. Correlation Analysis of the Markers

**Figure 1:**
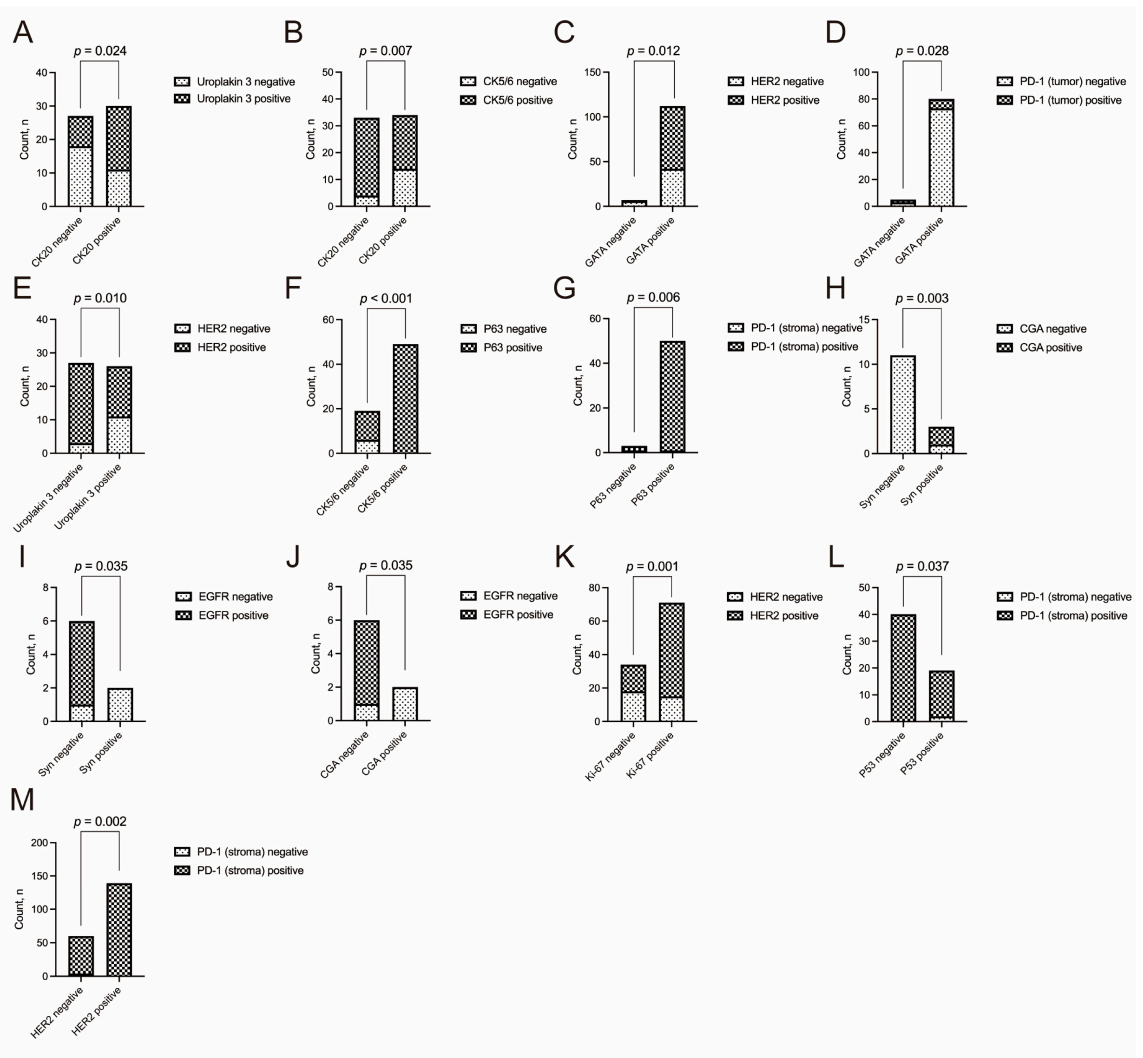
Correlation analyses of the marker expression. Positive counts of (**A**) Uroplakin 3 and (**B**) CK5/6 in CK20-negative/positive patients; (**C**) Her2 and (**D**) PD-1 (tumor) in GATA3-negative/positive patients; (**E**) Her2 in Uroplakin 3-negative/positive patients; (**F**) P63 in CK5/6-negative/positive patients; (**G**) PD-1 (stroma) in P63-negative/positive patients; (**H**) CGA and (**I**) EGFR in Syn-negative/positive patients; (**J**) EGFR in CGA-negative/positive patients; (**K**) Her2 in Ki-67-negative/positive patients; (**L**) PD-1 (stroma) in P53-negative/positive patients; (**M**) PD-1 (stroma) in Her2-negative/positive patients.

We analyzed whether the expression of these markers was correlated. The results showed that the positive rate of Uroplakin 3 was significantly higher in CK20-positive patients than in CK20-negative patients (*p* = 0.024), whereas the positive rate of CK5/6 was significantly lower in CK20-positive patients than in CK20-negative patients (*p* = 0.007). The negative rates of Her2 and PD-1 (tumor) were both significantly higher in GATA3-positive patients than in GATA3-negative patients (*p* = 0.012 and *p* = 0.028, respectively). The positive rate of Her2 was significantly lower in Uroplakin 3-positive patients than in Uroplakin 3-negative patients (*p* = 0.010). The positive rate of P63 was significantly higher in CK5/6-positive patients than in CK5/6-negative patients (*p* < 0.001). The positive rate of PD-1 (stroma) was significantly higher in P63-positive patients than in P63-negative patients (*p* = 0.006). The positive rate of CGA was significantly higher in Syn-positive patients than in Syn-negative patients (*p* = 0.003), whereas the positive rate of EGFR was significantly lower in Syn-positive patients than in Syn-negative patients (*p* = 0.035). The positive rate of EGFR was significantly lower in CGA-positive patients than in CGA-negative patients (*p* = 0.035). The positive rate of Her2 was significantly higher in Ki67-positive patients than in Ki67-negative patients (*p* = 0.001). The positive rate of PD-1 (stroma) was significantly lower in P53-positive patients than in P53-negative patients (*p* = 0.037). The positive rate of PD-1 (stroma) was significantly higher in Her2-positive patients than in Her2-negative patients (*p* = 0.002). The bar charts illustrating the correlations among these markers are shown in Figure 1.

### 3.3. Comparison of the Expression of the Markers Between Different Groups

We grouped patients according to pathological grade and muscle invasion status to compare differences in marker expression between groups. When grouped by pathological grade, we found significant differences in the expression of Ki67, Her2, and PD-L1 between low-grade and high-grade groups. The proportion of high-grade patients was significantly higher among Ki67-positive patients than among Ki67-negative patients (93.59% vs. 6.41%, *p* < 0.001). The proportion of high-grade patients was significantly higher among Her2-positive patients than among Her2-negative patients (86.92% vs. 13.08%, *p* < 0.001). The proportion of high-grade patients was significantly higher among PD-L1-positive patients than among PD-L1-negative patients (100% vs. 0%, *p* = 0.001). No significant differences were observed in the positive rates of the remaining pathological markers between high-grade and low-grade bladder cancer patients. When grouped by muscle invasion status into the non-muscle-invasive bladder cancer (NMIBC) group and the muscle-invasive bladder cancer (MIBC) group, we found that the proportion of MIBC patients was significantly higher among CK20-positive patients than among CK20-negative patients (51.79% vs. 48.21%, *p* = 0.008). The proportion of MIBC patients was significantly higher among Uroplakin 3-positive patients than among Uroplakin 3-negative patients (61.76% vs. 38.24%, *p* = 0.029). The proportion of MIBC patients was significantly lower among patients with positive stromal PD-1 than among those with negative stromal PD-1 (27.18% vs. 72.82%, *p* = 0.001). No significant differences were found in the positive rates of the remaining markers between MIBC and NMIBC patients. Comparison of the expression of the markers between different groups is presented in Table 2.

### 3.4. Diagnostic Value of Markers for High-Grade Disease and MIBC

In predicting high-grade disease, univariate analysis revealed that Ki67 and Her2 had diagnostic value. High Ki67 expression might be a strong predictor for distinguishing high-grade from low-grade disease, and Ki67-positive patients had a 17-fold increased risk of high-grade disease compared with Ki67-negative patients (OR = 17.381, *p* < 0.001). Her2 positivity might also be significantly associated with high-grade disease, and Her2-positive patients had a 2.5-fold increased risk of having high-grade disease compared with Her2-negative patients (OR = 2.513, *p* = 0.012). Multivariate analysis (including Ki67 and Her2) further confirmed that both Ki67 (*p* < 0.001) and Her2 (*p* = 0.001) might have association with high-grade disease. The diagnostic value of the markers for high-grade disease is shown in Table 3.

In predicting MIBC, univariate analysis showed that CK20 negativity (OR = 0.302, *p* = 0.010) and Uroplakin 3 negativity (OR = 0.258, *p* = 0.036) were significantly associated with an increased risk of MIBC, whereas the remaining markers showed no statistical significance. However, when CK20 and Uroplakin 3 were included in multivariate analysis, the effects of both markers attenuated and became non-significant (CK20, OR = 0.519, *p* = 0.352; Uroplakin 3, OR = 0.403, *p* = 0.194). The diagnostic value of the markers for MIBC is shown in Table 4.

### 3.5. Prognostic Value of Markers for Survival

**Figure 2:**
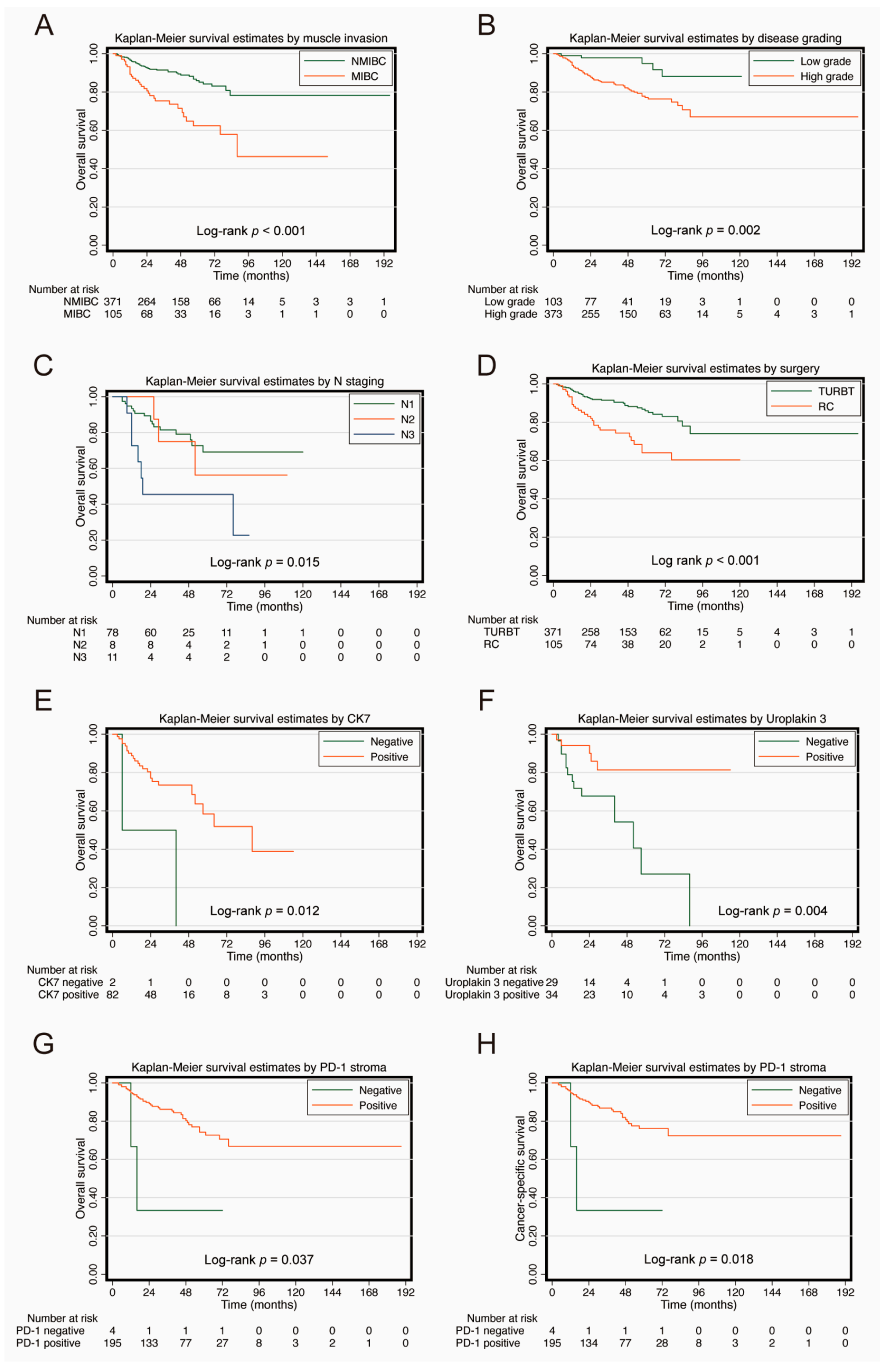
Comparison of survival by pathological characteristics and markers. Comparison of the overall survival by (**A**) muscle invasion, (**B**) disease grading, (**C**) N staging, (**D**) surgery, (**E**) CK7 expression, (**F**) Uroplakin3 expression and (**G**) PD-1 (stroma) expression; (**H**) Comparison of the cancer-specific survival by PD-1 (stroma) expression.

Kaplan–Meier survival analysis showed that, in addition to the well-established factors of muscle invasion (*p* < 0.001), pathological grade (*p* = 0.002), lymph node metastasis (*p* = 0.015), and surgical approach (*p* < 0.001), which all had significant effects on overall survival (OS), CK7 positivity (*p* = 0.012), Uroplakin 3 positivity (*p* = 0.004), and PD-1 (stroma) positivity (*p* = 0.037) were all associated with significantly better OS compared with negative groups. Patients with positive PD-1 (stroma) expression also demonstrated significantly better cancer-specific survival (CSS) than those with negative expression (*p* = 0.018). Comparisons of survival by pathological characteristics and markers are shown in Figure 2.

**Figure 3:**
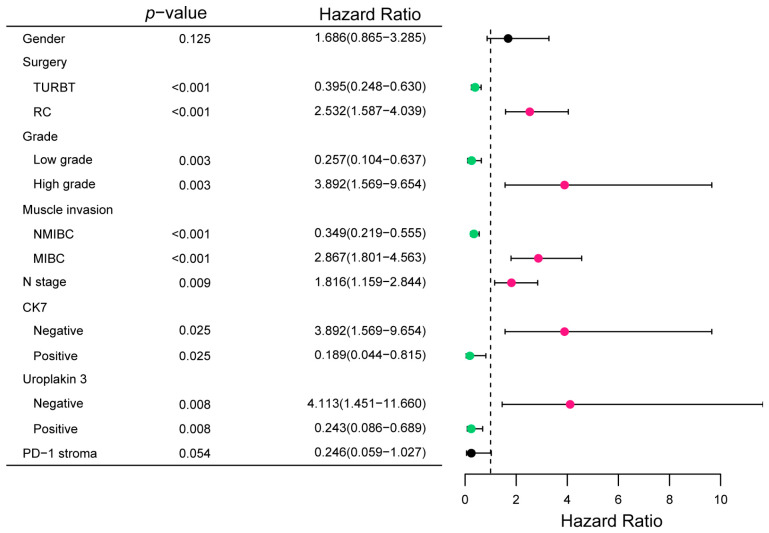
Forest plot of Cox regression analyses of overall survival. RC (*p* < 0.001), high-grade disease (*p* = 0.003), MIBC (*p* < 0.001), and lymph node metastasis (*p* = 0.009) all significantly increased the risk of survival events. Patients who were CK7 negative (*p* = 0.025) and Uroplakin 3 negative (*p* = 0.008) also had a significantly increased risk of survival events. Green dots indicate that the risk of the cohort is significantly lower than that of the control group, purple dots indicate that the risk of the cohort is significantly higher than that of the control group, and black dots indicate that the risk of the cohort is not significantly different from that of the control group.

Univariable Cox regression analyses revealed that among clinical factors, surgical approach (RC vs. TURBT, HR = 2.532, *p* < 0.001), muscle invasion (MIBC vs. NMIBC, HR = 2.867, *p* < 0.001), and lymph node metastasis (positive vs. negative, HR = 1.816, *p* = 0.009) were all significantly associated with increased risk of events. CK7 positivity (HR = 0.189, *p* = 0.025) and Uroplakin 3 positivity (HR = 0.243, *p* = 0.008) significantly reduced the risk of events, both of which might be protective factors; the remaining markers showed no prognostic value for survival. As the populations corresponding to the different markers differed, we did not perform a multivariable Cox analysis. The prognostic value of the markers for OS by Cox regression analyses is presented in Table 5. The forest plot of Cox regression analyses of OS is shown in Figure 3.

## 4. Discussion

We systematically and retrospectively analyzed the clinicopathological data and various clinically used immunohistochemical markers from 477 patients to evaluate the value of these markers in predicting high-grade disease, MIBC and survival in bladder cancer.

In predicting high-grade disease, we found that the proportions of Ki67-positive and Her2-positive patients were significantly increased among high-grade patients, and logistic regression analyses further confirmed the association between Ki67, Her2 and high-grade disease. Ki67 expression level directly reflects tumor cell proliferative activity and is a core immunohistochemical marker for assessing cellular proliferation [12,13]. Our findings further confirm the important value of Ki67 in predicting high-grade disease. Her2 is a member of the epidermal growth factor receptor (EGFR) family that can activate downstream signaling pathways to promote tumor cell proliferation and invasion while inhibiting apoptosis [14]. Current research generally supports that Her2 overexpression is closely associated with high-grade bladder cancer [15], and our findings are consistent with this.

Referring to MIBC, we found that none of the markers have diagnostic value, with only univariate logistic analysis suggesting that Uroplakin 3 and CK20 might have a potential association with MIBC. This conclusion differs from some studies; Tsumura et al. found that Uroplakin 3 levels were associated with muscle-invasive status [16], and Sikic et al. considered CK20 as a predictor of MIBC [17]. It should be noted that for Uroplakin 3, both Fisher’s exact test (*p* = 0.029) and univariate logistic regression (*p* = 0.036) identified a significant association between Uroplakin 3 level and MIBC; however, the significance of Uroplakin 3 disappeared after multivariate logistic regression (*p* = 0.194). A similar pattern was observed for CK20. Therefore, we are inclined to attribute this to a statistical issue caused by insufficient sample size rather than a lack of diagnostic value of Uroplakin 3 and CK20 in MIBC. Considering this, the diagnostic value of these two markers in MIBC might require further clarification through studies with larger sample sizes.

Nevertheless, we believe that the clinical significance of the above findings might be relatively limited, as obtaining the expression levels of these markers requires access to the patient’s bladder cancer tissue, and once tissue is obtained, disease grade and stage can be easily determined. These results primarily suggest that proteins such as Ki67, Her2, CK20, and Uroplakin 3 might play certain roles in bladder cancer progression, providing some direction for future research.

In predicting survival, we found that both CK7 and Uroplakin 3 might be protective factors for OS; CK7 positivity (HR = 0.189, *p* = 0.025) and Uroplakin 3 positivity (HR = 0.243, *p* = 0.008) significantly reduced the risk of events. However, considering there were only 2 CK7-negative patients and the remaining 82 (97.6%) patients were CK7-positive, the sample size might be insufficient to demonstrate the prognostic value of CK7 for OS. In contrast, both Uroplakin 3-negative and Uroplakin 3-positive groups had adequate sample sizes (34 positive and 29 negative patients in total), which should be sufficient to demonstrate the prognostic value of Uroplakin 3 for OS. We did not analyze NMIBC and MIBC separately because we aimed to evaluate the prognostic value of these markers across the overall bladder cancer population—that is, their ability to provide prognostic information without prior knowledge of whether the tumor was muscle-invasive or not.

Uroplakin 3 is a transmembrane glycoprotein with a molecular weight of approximately 25 kDa that assembles into tetramers and hexamers through heterodimer formation, ultimately constituting the highly ordered asymmetric unit membrane (AUM) structure [18]. Uroplakin 3 is essential for maintaining urothelial integrity, barrier function, and mechanical strength. Loss of Uroplakin 3 protein can lead to disruption of the AUM structure, while tumor cells may acquire a more aggressive basal-like or mesenchymal-like phenotype due to loss of the terminally differentiated umbrella cell phenotype, making it easier to breach the basement membrane and invade the muscle layer, ultimately progressing to MIBC [18]. Furthermore, Uroplakin 3 negativity is usually accompanied by complete silencing of the urothelial differentiation program and activation of basal cell differentiation pathways, Epithelial–Mesenchymal Transition (EMT) pathways, or neuroendocrine differentiation pathways; consequently, Uroplakin 3-negative disease often corresponds to more aggressive subtypes such as basal, neuroendocrine, or mesenchymal-like types [19,20]. Some studies have also found that Uroplakin 3-negative tumor cells, due to complete dedifferentiation and EMT activation, possess high environmental adaptability, enabling survival in hypoxic and nutrient-deficient microenvironments, and can breach tissue barriers to colonize distant organs and form metastatic foci [21]. Matsumoto et al. studied 92 bladder cancer patients who underwent radical cystectomy and found that patients with loss of Uroplakin 3 expression had significantly worse CSS [22], which is consistent with our conclusions. This implies that immunohistochemical staining for Uroplakin 3 can further stratify bladder cancer patients into two subtypes: Uroplakin 3-positive and Uroplakin 3-negative, with the Uroplakin 3-negative subtype being associated with worse OS.

This study has several limitations. First, the statistical power was limited for CK7 with only 2 negative cases, making it impossible to reliably assess its prognostic value, and the overall sample size needs to be expanded to further validate the conclusions. Second, as a single-center retrospective study, there is a risk of selection bias, and the short follow-up time may affect the accuracy of the survival analyses. Third, the clinical applicability is limited, as tumor tissue must first be obtained to detect markers, by which time pathological stage and grade are usually already determined, creating a logical paradox regarding their diagnostic significance. Fourth, as this was a retrospective study, all data were derived from the patients’ formal pathology reports, and no additional immunohistochemical staining was performed. Consequently, the sample sizes for specific markers were largely determined by the pathology department’s assessment of the necessity for such testing. This implies that the testing of certain markers was likely based on clinical indications, introducing significant selection bias. Given this context, we aimed to include all eligible patients in the study and did not conduct sample size calculations. However, it is evident that for certain markers, including P40 (15 patients), Syn (15 patients), CD56 (13 patients), CGA (14 patients), Vimentin (7 patients), and LCA (3 patients), the sample sizes are likely insufficient to support robust conclusions about them. Fifth, next-generation sequencing (NGS) could have significantly enhanced the comprehensiveness of this study; however, due to funding constraints, we did not employ NGS. This represents one of the study’s primary limitations and is an area we intend to address in future work.

However, most immunohistochemical markers remain research tools, and their clinical value, including diagnostic and prognostic value, needs further clarification, while HER2 testing has a specific role in selecting patients for HER2-targeted therapy in advanced disease. In summary, our conclusions are only preliminary and require further validation through larger-scale studies in the future.

## 5. Conclusions

The results suggest that Ki67 positivity and Her2 positivity might be associated with high-grade bladder cancer, and Uroplakin 3 positivity might have prognostic value for better OS. However, these conclusions require further clarification through larger-scale studies in the future.

## Figures and Tables

**Table 1:** Patients’ clinicopathologic characteristics.

Characteristics	Total
Patients, n	477
Age, years	67.405 ± 11.053
Gender, n (%)	
Male	378 (79.25)
Female	99 (20.75)
Surgery	
TURBT	371 (77.78)
RC	106 (22.22)
Pathological grade, n (%)	
Low grade	103 (21.59)
High grade	374 (78.41)
Pathological T staging, n (%)	
Tis	6 (1.26)
Ta	210 (44.03)
T1	155 (32.49)
T2	57 (11.95)
T3	38 (7.97)
T4	11 (2.31)
Muscle invasion, n (%)	
No (Tis + Ta + T1)	371 (77.78)
Yes (T2 + T3 + T4)	106 (22.22)
Pathological N staging, n (%)	
N0	78 (80.41)
N1	8 (8.25)
N2	11 (11.34)
Event	
Yes	73 (15.30)
Time, month	27.658 ± 22.004
No	404 (84.70)

TURBT, transurethral resection of bladder tumor; RC, radical cystectomy.

**Table 2:** Comparison of the expression of the markers between different groups.

Markers	Patients, n	Positive, n (%)	Grade, n (%)		*p*-Value	Muscle Invasion, n (%)		*p*-Value
			Low	High		No (NMIBC)	Yes (MIBC)	
**Epithelial/urothelial markers** confirm urothelial origin								
CK7	84	82 (97.62)	10 (12.20)	72 (87.80)	0.599	26 (31.71)	56 (68.29)	0.338
CK20	97	56 (57.73)	9 (16.07)	47 (83.93)	0.196	27 (48.21)	29 (51.79)	0.008
GATA3	128	120 (93.75)	14 (11.67)	106 (88.33)	0.072	49 (40.83)	71 (59.17)	0.230
Uroplakin 3	63	34 (53.97)	3 (8.82)	31 (91.18)	0.383	13 (38.24)	21 (61.76)	0.029
**Basal/squamous differentiation markers** indicate squamous differentiation or basal cell expression								
CK5/6	87	63 (72.41)	8 (12.70)	55 (87.30)	0.243	24 (38.10)	39 (61.90)	0.959
P40	15	12 (80.00)	0	12 (100)	-	4 (33.33)	8 (66.67)	0.243
P63	97	89 (91.75)	9 (10.11)	80 (89.89)	0.345	34 (38.20)	55 (61.80)	0.147
**Neuroendocrine markers** diagnose small cell carcinoma								
Syn	15	3 (20.00)	0	3 (100)	-	1 (33.33)	2 (66.67)	0.770
CD56	13	3 (23.08)	0	3 (100)	-	1 (33.33)	2 (66.67)	0.326
CGA	14	2 (14.29)	0	2 (100)	-	0	2 (100)	0.425
**Mesenchymal/melanocytic markers** Rule out sarcomatoid carcinoma or metastatic melanoma								
Vimentin	7	2 (28.57)	1 (50)	1 (50)	0.088	1 (50)	1 (50)	0.809
**Lymphohematopoietic markers** exclude lymphoma								
LCA (CD45)	3	1 (33.33)	0	1 (100)	-	0	1 (100)	-
**Proliferation/Prognostic markers** Assess aggressiveness and prognosis								
Ki67	124	78 (62.90)	5 (6.41)	73 (93.59)	<0.001	61 (78.21)	17 (21.79)	0.785
P53	115	39 (33.91)	5 (12.82)	34 (87.18)	0.444	23 (58.97)	16 (41.03)	0.564
**Therapeutic targets markers** Potential for targeted therapy								
Her2	329	214 (65.05)	28 (13.08)	186 (86.92)	<0.001	160 (74.77)	54 (25.23)	0.397
EGFR	53	48 (90.57)	9 (18.75)	39 (81.25)	0.288	25 (52.08)	23 (47.92)	0.172
**Immune checkpoint markers** predict response to immunotherapy								
PD-L1	190	34 (17.89)	0	34 (100)	0.001	21 (61.76)	13 (38.24)	0.248
PD-1 (tumor)	225	37 (16.44)	6 (16.22)	31 (83.78)	0.575	26 (70.27)	11 (29.73)	0.843
PD-1 (stroma)	199	195 (97.99)	34 (17.44)	161 (82.56)	0.359	142 (72.82)	53 (27.18)	0.001
**Other** diagnoses: rare NTRK fusion tumors								
Pan-TRK	82	3 (3.66)	1 (33.33)	2 (66.67)	0.492	3 (100)	0	0.180

NMIBC, non-muscle-invasive bladder cancer; MIBC, muscle-invasive bladder cancer. “Patients” refers to the number of patients who underwent immunohistochemical staining for the marker (since the data is directly quoted from the pathology report, some patients did not undergo immunohistochemical staining for a specific marker). “Positive” refers to the number and proportion of patients who tested positive among those who underwent immunohistochemical staining for the marker.

**Table 3:** Diagnostic value of the markers for high-grade disease.

Markers	Odds Ratio (Positive vs. Negative)	Standard Error	*z*-Value	*p*-Value	95% CI	
					LL	UL
**Univariable**						
CK20	0.412	0.289	−1.260	0.207	0.104	1.630
GATA3	4.543	3.560	1.930	0.053	0.978	21.107
Uroplakin 3	0.369	0.437	−0.840	0.400	0.036	3.756
CK5/6	0.299	0.326	−1.110	0.268	0.035	2.528
Ki67	17.381	9.541	5.200	<0.001	5.927	50.970
P53	1.535	0.864	0.760	0.446	0.509	4.629
Her2	2.513	0.919	2.520	0.012	1.227	5.145
PD-1 (tumor)	1.309	0.630	0.560	0.576	0.509	3.364
Pan-TRK	0.431	0.543	−0.670	0.504	0.036	5.087
CK7	0.567	0.677	−0.480	0.634	0.055	5.883
**Multivariable**						
Ki67	12.799	8.573	3.810	<0.001	3.444	47.567
Her2	9.349	6.072	3.440	0.001	2.617	33.392

95% CI, 95% confidence interval; LL, lower limit; UL, upper limit.

**Table 4:** Diagnostic value of the markers for MIBC.

Markers	Odds Ratio (Positive vs. Negative)	Standard Error	*z*-Value	*p*-Value	95% CI	
					LL	UL
**Univariable**						
CK20	0.302	0.140	−2.590	0.010	0.122	0.748
GATA3	2.415	1.820	1.170	0.242	0.551	10.576
Uroplakin 3	0.258	0.166	−2.100	0.036	0.073	0.913
CK5/6	0.975	0.483	−0.050	0.959	0.369	2.573
P63	0.231	0.252	−1.340	0.179	0.027	1.961
Syn	0.667	0.930	−0.290	0.771	0.043	10.253
CD56	0.222	0.359	−0.930	0.352	0.009	5.275
Vimentin	0.667	1.122	−0.240	0.810	0.025	18.059
Ki67	0.887	0.391	−0.270	0.785	0.373	2.106
P53	1.262	0.510	0.580	0.564	0.572	2.788
Her2	1.296	0.520	0.650	0.518	0.590	2.843
EGFR	0.230	0.266	−1.270	0.203	0.024	2.211
PD-L1	1.576	0.623	1.150	0.250	0.726	3.419
PD-1 (tumor)	0.925	0.363	−0.200	0.843	0.429	1.997
CK7	0.278	0.267	−1.330	0.182	0.042	1.824
**Multivariable**						
CK20	0.519	0.365	−0.930	0.352	0.131	2.062
Uroplakin 3	0.403	0.282	−1.300	0.194	0.102	1.588

MIBC, muscle-invasion bladder cancer; 95% CI, 95% confidence interval; LL, lower limit; UL, upper limit.

**Table 5:** Prognostic value of the markers for overall survival by univariable Cox regression analyses.

	Patients, n	Overall Events Observed (Expected)		Hazard Ratio (Positive vs. Negative)	95% CI		Standard Error	*p*-Value
		Negative Marker	Positive Marker		LL	UL		
CK7	84	2 (0.41)	24 (25.59)	0.189	0.044	0.815	0.141	0.025
CK20	97	13 (9.83)	11 (14.17)	0.586	0.262	1.309	0.240	0.193
GATA3	128	1 (1.74)	29 (28.26)	1.790	0.244	13.163	1.822	0.567
Uroplakin 3	63	13 (7.11)	5 (10.89)	0.243	0.086	0.689	0.129	0.008
CK5/6	87	7 (5.98)	15 (16.02)	0.799	0.325	1.962	0.366	0.624
P40	15	1 (1.00)	5 (5.00)	0.996	0.114	8.707	1.102	0.997
P63	97	3 (1.37)	20 (21.63)	0.415	0.121	1.417	0.260	0.160
Syn	15	3 (3.28)	1 (0.72)	1.513	0.156	14.652	1.752	0.721
CD56	13	3 (3.35)	1 (0.65)	1.741	0.176	17.185	2.034	0.635
CGA	14	3 (3.64)	1 (0.36)	3.766	0.336	42.220	4.644	0.282
Vimentin	7	2 (2.35)	1 (0.65)	1.818	0.161	20.510	2.247	0.629
Ki67	124	7 (6.99)	11 (11.01)	0.998	0.386	2.583	0.484	0.997
P53	115	14 (16.17)	10 (7.83)	1.477	0.656	3.327	0.612	0.346
Her2	329	18 (20.68)	41 (38.32)	2.878	0.899	9.209	1.708	0.075
EGFR	53	2 (1.22)	12 (12.78)	0.570	0.127	2.561	0.437	0.464
PD-L1	190	30 (29.36)	6 (6.64)	0.883	0.367	2.127	0.396	0.782
PD-1 (tumor)	225	33 (30.76)	4 (6.24)	0.597	0.211	1.686	0.316	0.330
PD-1 (stroma)	199	2 (0.52)	38 (39.48)	0.246	0.059	1.027	0.179	0.054

95% CI, 95% confidence interval; LL, lower limit; UL, upper limit.

## Data Availability

All the data sets generated in this study are available on reasonable request to the corresponding author.

## Abbreviations

The following abbreviations are used in this manuscript:

AJCC	American Joint Committee on Cancer
CI	Confidence Interval
CPS	Combined Positive Score
CSS	Cancer-Specific Survival
IHC	Immunohistochemical
LL	Lower Limit
MIBC	Muscle-Invasive Bladder Cancer
NMIBC	Non-Muscle-Invasive Bladder Cancer
OR	Odds Ratio
OS	Overall Survival
RC	Radical Cystectomy
SD	Standard Deviation
TURBT	Transurethral Resection of Bladder Tumor
UL	Upper Limit
